# Pentraxin-3 to better delineate necrotizing soft tissue infection: not really!

**DOI:** 10.1186/s13054-016-1319-0

**Published:** 2016-06-17

**Authors:** Patrick M. Honore, Herbert D. Spapen

**Affiliations:** ICU Department, Universitair Ziekenhuis Brussel, Vrije Universiteit Brussel, 101 Laarbeeklaan, 1090 Jette Brussels, Belgium

Necrotizing soft tissue infection (NSTI) is a devastating condition with high morbidity and a dismal prognosis. Timely and adequate surgery and early aggressive treatment of associated sepsis are imperative to improve survival [1–3]. Pentraxin-3 (PTX3) is a glycoprotein released by endothelial and inflammatory cells upon stimulation by cytokines and endotoxins. In contrast with C-reactive protein (CRP) which is produced in the liver in response to systemic inflammation, PTX3 is thought to better reflect local vascular inflammation and bacterial load [2]. PTX3 assessment might thus be a more appropriate marker of severity and prognosis of NSTI. This was corroborated by Hansen et al. [1], who reported a significant association between high baseline PTX3 levels and occurrence of septic shock, amputation, dialysis need, and risk of death in a large cohort of patients with NSTI. PTX3 also performed better than the “classical” inflammatory markers CRP and procalcitonin (PCT).

These findings are interesting but mandate careful reflection. First, they are not unexpected. Measurement of PTX3 has been shown to improve patient risk assessment and persistently elevated levels during evolving sepsis may contribute to tissue damage and predict poor patient outcome [2]. Second, when looking closely at the study results, PTX3 levels appeared to be fairly in line with PCT concentrations for assessing NSTI-associated morbidity and mortality. PCT also outperforms CRP in predicting severity of infection and organ dysfunction in sepsis [4] but is more readily available than PTX3. Finally, many patients with NSTI develop acute kidney injury, necessitating renal replacement therapy (RRT) (25 % of the patients studied by Hansen et al.!). PTX3 has a molecular weight of approximately 35 kDa [2] and thus in theory can be removed by RRT. Recently, Schilder et al. [5] demonstrated some adsorption but no elimination of PTX3 by convection across the system of continuous veno-venous hemofiltration (CVVH), resulting in unaltered plasma levels. Whether this is also relevant when CVVH is performed at higher convection flux or with different types of dialysis membranes (especially highly adsorptive) remains to be determined.

## Abbreviations

CRP: C-reactive protein
CVVH: continuous veno-venous hemofiltration
NSTI: necrotizing soft tissue infection
PCT: procalcitonin
PTX3: pentraxin-3
RRT: renal replacement therapy
