# Supplementary material for: Pentraxin-3 to better delineate necrotizing soft tissue infection: not really!
Source: Crit Care. 2016 Jun 17;20:173. doi: 10.1186/s13054-016-1319-0 (PMC4911691; doi:10.1186/s13054-016-1319-0)
Supplement: Related texts

# Reply: Authors’ response

**Authors:** Marco Bo Hansen, Lars Simon Rasmussen, Peter Garred, Daniel Bidstrup, Martin Bruun Madsen, Ole Hyldegaard

The authors would like to thank Dr Honore and Dr Spapen for their interest in our article. In their letter, Honore and Spapen propose three important points for consideration. First, they address the observation that PTX3 has been shown to improve risk assessment in septic patients. We agree that previous, but few, studies have investigated PTX3 in patients with sepsis. However, PTX3 has not been investigated in patients with NSTI. This is important to address because the immunological processes are probably different during infections that cause massive necrosis, as in the case of NSTI. Since NSTI is an uncommon disease, treatment and surveillance procedures in the ICU rely, primarily, on knowledge obtained from studies on septic patients. Thus, it is important to establish solid evidence for the treatment strategies in patients with NSTI as well, which also includes investigating the different aspects of the immunological response. In addition, suspicion of NSTI is currently based on the patient’s clinical presentation, making it interesting to investigate the predicting abilities of new biomarkers, such as PTX3.

Second, they underline that both PTX3 and PCT are good predictors of morbidity and mortality. We agree with this and the fact that both biomarkers outperform CRP. Our results suggest that PTX3 can be considered a reliable marker of infections on equal terms to PCT in patients with NSTI. However, the PTX3 level was significantly higher in patients needing amputation. This was not the case for CRP or PCT. PTX3 might therefore be useful to identify different subgroups of patients from PCT.

Third, Honore and Spapen speculate whether PTX3 can be removed by RRT, thus altering the levels in the study cohort. Despite PTX3 having a molecular weight of 45 kDa as a monomer, it forms an octamer in plasma composed of two covalently linked tetramers with a weight above 400 kDa [6–8]. It is therefore unlikely that PTX3 will be removed by RRT. Moreover, the baseline samples were taken upon admission and before initiation of RRT in the vast majority of patients.
